# Gendered time use among specializing medical doctors at Makerere University, Uganda: a cross-sectional study

**DOI:** 10.1080/16549716.2026.2636410

**Published:** 2026-03-03

**Authors:** Angela N. Kisakye, Suzanne Namusoke Kiwanuka, Isabel Goicolea, Ida Linander, Helene Johansson

**Affiliations:** a Department of Epidemiology and Global Health, Umeå International School of Public Health, Umeå University, Umeå, Sweden; b Department of Health Policy Planning and Management, Makerere University School of Public Health, Kampala, Uganda

**Keywords:** Time use, gender differences, Uganda, health workforce, unpaid domestic work

## Abstract

**Background:**

Previous research shows that gendered differences in time use unfairly impact women’s career advancement and influence workforce management. Despite a growing body of literature on gendered time use, the topic has not been well documented among specializing medical doctors in sub-Saharan African countries, including Uganda.

**Objectives:**

This study was conducted among specialist medical doctors at Makerere University to: (i) analyze gendered differences in time use for paid and unpaid activities and (ii) assess whether parenting influences time use.

**Methods:**

The study sample comprised 244 medical doctors pursuing graduate specialist programs in 2024. The data collection, which utilized a self-administered questionnaire, assessed socio-demographic factors and self-reported time spent on paid work, unpaid domestic work, unpaid care for household members, studying, socializing, and leisure activities. Quantile regression analysis, with 95% confidence intervals, was used to compare median differences in reported time use across various activities for men and women.

**Results:**

Compared to men, women reported spending more time on unpaid domestic work (2 vs 1 h/day: 95% CI: 0.6, 1.4) and less time on leisure activities (4 vs 7 h/week: 95% CI: −5.3, −0.8). Women with children spent more time on paid work than their male counterparts. Women with children reported spending half a day more on paid work and an additional hour on unpaid domestic work compared to men with children.

**Conclusion:**

This gender imbalance in time use could negatively impact the career progression and well-being of female doctors and further reinforce gender inequalities in the medical workforce in Uganda.

## Paper context

- **Main finding:** Female specialist medical doctors in Uganda spend significantly more time on unpaid domestic work and less on leisure and study than their male counterparts, with parenting responsibilities further widening these disparities.
- **Added Knowledge:** This study provides insights from a low-income country context on how gendered time-use patterns intersect with parenting to influence professional and personal time allocation among specialist doctors. This topic has not been explored in sub-Saharan Africa.
- **Global health impact for policy and action:** This study provides evidence to inform gender-responsive policies, flexible work arrangements, and equitable career pathways.

## Background

Gendered time use refers to how men and women allocate their time differently across daily activities, both paid and unpaid [1]. Time-use studies conducted in high-income countries have examined the causes and consequences of the gender gap in paid and unpaid work [2–4]. The findings consistently show that, regardless of socio-economic status, women spend more time than men on unpaid caregiving and household tasks – a factor which has been linked to workplace stress, fewer promotion opportunities, lower job satisfaction, and delays in pursuing further education and professional development [5–7]. Women are also more likely to work part-time, yet they generally have less ‘leisure time’ [8,9]. Studies conducted in various settings show that, unlike men, women experience a ‘double burden’ (the combined responsibility of paid employment and unpaid domestic and care work) – a contributing factor to poor health and low well-being [10,11].

While gender inequalities in the labour market are a global issue, the manifestations vary across settings [10]. Family support can help in some circumstances, yet inadequate and unaffordable childcare limits women’s workforce participation [12]. Some countries have short-term parental leave policies, but gender bias in uptake can reinforce rather than mitigate existing inequalities [13,14]. Based on the gender equality seal for public institutions, gender responsive institutions should ensure that work–life balance is actively promoted, including fostering co-responsibility between men and women for unpaid domestic and care work [15]. There have been advances in gender equality across different contexts aimed at ensuring inclusivity of men and women within the workplace, meeting gender targets in leadership and gender targets in supporting working parents. For instance, more countries are expanding parental leave, including policies that encourage paternity leave to ensure that there is equality in childcare. Workplaces are also embracing flexible working options such as remote work and flexible working hours to better accommodate working parents [16–18].

Gendered time-use inequalities are the result of deeply entrenched socio-cultural norms that define gender roles within households [5]. In sub-Saharan Africa and other regions such as Asia, inequalities in gendered time use are influenced by religious beliefs, institutional, and economic structures that prioritize men’s paid work [17,19]. Unconscious bias in health systems has also contributed to a lack of work–family balance policies that could potentially reduce this burden [20]. Policies aimed at balancing work–family life are important in managing and retaining human resources [21].

Despite a growing body of literature on gendered time use, the topic has not been well documented among (specializing) medical doctors in sub-Saharan African countries, including Uganda. Studies from other contexts, for instance, the USA and the United Kingdom, have documented how the constraints in time use for specializing women doctors negatively affect the time dedicated to study and mentorship programs, eventually influencing career progression and speciality choices [22,23]. For instance, women may prefer to undertake less time-intensive specialties perceived as more compatible with family responsibilities, such as paediatrics or public health, while men dominate surgical specialities that are time-intensive [24,25]. Within the Ugandan context, while studies on gendered time use among medical doctors have not been conducted, existing reports indicate that training institutions have rigid training schedules with no consideration for female caregiving roles, which may likely affect women’s career progression and specialty distribution [26–29]. It is important as well to mention that in the Ugandan context, highly educated women, such as medical doctors, can afford house helpers who usually take on some of the domestic work, which allows women to pursue their careers or studies. In addition, within the Ugandan context, grandparents often take on childcare and domestic activities in many households, enabling working parents to spend time at their workplace [30,31].

Previous research has linked *time poverty* – often resulting from the burden of unpaid domestic activities – to reduced opportunities for women to pursue and/or complete further education. In the health system, this can lead to unfair gender disparity in promotions and pay and impact service delivery and health care outcomes [32,33]. The double burden of paid and unpaid work responsibilities has been associated with chronic stress and job dissatisfaction among women [34,35]. In Uganda, Makerere University adapted the UNDP gender equality seal as the first public institution with targets towards gender inclusivity. For instance, the university has enhanced female enrolment by awarding them an extra 1.5 points during admission to the university [36]. Recent milestones in achieving gender equality in Makerere University have included the establishment of a child care center to allow the provision of daycare and breastfeeding for female staff and students to achieve their education and professional milestones without interruption [37]. These developments signify the recognition that intentional systemic changes have to be made to foster gender parity in educational pursuits and career progress. However, research that assesses gendered time use within this context of gender equality policies development is lacking.

Understanding gender inequalities in the health sector is important not only for workforce planning, task allocation and capacity-building but for promoting gender equality more broadly. To fill this knowledge gap, the current study, which was conducted among specialist medical doctors at Makerere University School of Medicine in Uganda, aims to: (i) analyze gendered differences in time use for paid and unpaid activities and (ii) assess whether parenting influences time use.

## Methods

### Study design and setting

In Uganda, medical doctors are trained at seven public and six private universities. This cross-sectional study was conducted between September 2024 and January 2025 at the country’s oldest and largest medical training institution – Makerere University College of Health Sciences, School of Medicine, a public university established in 1924 and located in Kampala [38]. Participants were selected from 12 postgraduate specialization programs. The other six universities in Uganda were not considered because they enrol smaller cohorts of students and do not offer all specializations [39].

### Ethics approval

Ethics approval was obtained from Makerere University School of Public Health Research Ethics Committee (IRB NO. SPH-2024–622) and the Uganda National Council for Science and Technology (UNCST). This study is in accordance with the principles of the Declaration of Helsinki.

### Sample size

The total number of postgraduate medical doctors enrolled in specialist training at Makerere University at the time of data collection was 441, from which we invited 369 to participate.

### Recruitment

Participants’ details were obtained from the Health Systems Management course coordination lists at Makerere University. Course administrators made these available to the study team. Class representatives who were identified from the class lists were consulted by phone to identify suitable times for the study team to meet potential participants in their lecture rooms. Data collection was scheduled during breaks from lectures or hospital ward activities to minimize disruption. In addition, the Principal Investigator (PI) contacted participants who were not available during the study breaks via phone and email to explain the purpose of the study and how the data were to be collected.

Two research assistants, both with bachelor’s degrees in environmental health sciences, were recruited. Their training focused on ensuring that they were familiar with the questionnaire and both electronic and paper-based data collection procedures.

Written informed consent was obtained from all participants prior to the commencement of data collection. The questionnaire did not contain the name of the participant, and it is not possible to trace back participants based on the questionnaires. This was done to ensure confidentiality.

### Data collection

Data were collected from September 2024 to January 2025 using a self-administered questionnaire that had questions adapted from the Zambia Labour Force Survey, which was conducted in 2018 [40] and from a questionnaire that measured time use in developing settings by Seymour *et al* [41]. The questionnaire, written in the English language, was pre-tested among Public Health students at Makerere University School of Public Health. Revisions were made to the questionnaire based on participants’ feedback. The measurements for leisure and rest were revised from hours per day to hours per week because leisure and rest are mainly concentrated over the weekend.

A total of 369 postgraduate medical specialist students were invited to participate in the survey. The final sample consisted of 244, leading to a response rate of 66%. Reasons for unavailability included hospital commitments (in theatres or wards), being on a gap year and declining to provide consent.

Participants were given a self-administered paper questionnaire during study breaks. The participants were met in their lecture rooms, and each participant was handed a questionnaire which they filled out for an average time of 40 min. Participants who preferred to complete the questionnaire electronically were provided with a digital version. The questionnaire was designed to capture how individuals allocate their time across various activities over the course of a day or week. This is in contrast to time diaries, which typically require detailed recordings made at short frequent intervals. The questionnaire also collected information on socio-demographic factors, as well as the time spent on paid work, unpaid work, unpaid domestic tasks, unpaid care, learning and studying, leisure, resting, and sleeping.

### Variables

The variables included in the analyses were informed by contextual factors that refer to regular daily and weekly activities. The dependent variable *time use captures* reported time spent on the activities listed in the questionnaire. The measurements are in days/week, h/day and h/week. See Table 1.

**Table 1.** Time use categories.

Variable	Question asked
**Time spent on paid work** This includes average work time (days/week) spent doing main and any other paid work	On average, how many days per week do you usually work? On average how many days do you spend on your other job?
**Time spent on unpaid domestic work** This was time (h/day) spent preparing meals and cleaning within the household.	On average, how much time per day do you spend on cleaning? On average, how much time per day do you spend on cooking?
**Time spent on unpaid care work for household members** Time (h/day) spent caring for young children, people with disabilities, the ill household members, and the elderly.	On average, how much time per day do you spend providing care, help, or assistance to household members aged 18 years and above due to disability? On average, how much time per day do you spend providing care, help or assistance to household members aged 18 years and above due to illness? On average, how much time per day do you spend looking after children aged 17 years or younger living in this household?
**Time spent on learning and studying** This refers to time (h/week) spent reading, studying, attending, or any other educational activities.	On average, how much time do you spend on attending school, carrying out any other type of studies, doing homework, and or going to the place where you study per week?
**Time spent on leisure** This was the time (h/week) spent socializing, eating out, going to bars, participating in sports or excercise.	On average, how much time per week do you spend socializing? On average, how much time per week do you spend on playing sports, exercising and outdoor activities, including playing football, walking the dog, going to the park? On average, how much time per week do you spend on eating out/going to the pub, including going to cafes, bars, restaurants and nightclubs?
**Time spent resting/sleeping** This was the time (h/week) spent sleeping/resting.	On average, how much time per week do you spend on resting, sleeping and relaxing?

Time reported for either main or secondary jobs was combined and analysed under the category *paid work*. Other jobs in our study referred to secondary professional engagements undertaken by the participants outside their primary workplace, for instance, working in private clinics and managing pharmacies. References to overtime were minimal, mainly because the respondents were students still in training, and all overtime was excluded from the analysis. Time spent on caregiving for young children, the elderly, the ill, or persons with disabilities was combined as *unpaid care work for household members*. Time spent on housework activities such as cooking and cleaning was categorized as *unpaid domestic work*. Time spent on educational activities was categorized as *learning and studying*; time spent on socializing, dining out, going to bars, participating in sports, and exercising was grouped under *leisure*. There were three dichotomized independent variables: (i) *gender* (female vs male); (ii) *marital status* (in union vs not in union) and (iii) *parental status* (have children vs do not have children). The variables that were considered as confounders were those that were significantly associated with the outcome based on the literature [42,43].

### Data analysis

The data were entered into Kobo Collect, cleaned using Microsoft Excel, and exported to STATA version 14 for analysis. Descriptive statistics were generated; percentages are given for categorical variables with means and standard deviations for age expressed as a continuous variable. Given that time-use variables were not normally distributed, medians and interquartile ranges (IQRs) were used as summary measures [44]. Quantile regression analysis was used to estimate median differences in time use across activity categories by gender, with 95% confidence intervals. Statistically significant associations were further adjusted for age (25–34 years vs 35–50 years), marital status (not in union vs in union) and parental status (have children vs do not have children) as potential confounders.

## Results

### Socio-demographic profile of respondents

Most participants (61.5%) were men. The mean age was 32 years (SD = 0.22). More than three-quarters (78.7%) were aged between 25 and 34 years, and approximately half (56.2%) were in a marital union (61.3% men and 47.9% women). Less than one in five (18.0%) were in their first year of specialization. Almost half (46.7%) were in their second year, 32.4% were in their third and 2.9% were in their fourth. Almost half of the women (48%) had children, while 39.4% of the men had children (Table 2).

**Table 2.** Participants’ socio-demographic characteristics.

Variables	Total	Men	Women
**Sex, n (%)**		150 (61.5%)	94 (38.5%)
**Age (years), mean (SD)**		33, SD (0.3)	31, SD (0.4)
**Age groups (years), n (%)**			
25–34	192 (78.7%)	115 (76.7%)	77 (81.9%)
35–50	52 (21.3%)	35 (23.3%)	17 (18.1%)
**Marital status, n (%)**			
Not in Union	107 (43.9%)	58 (38.7%)	49 (52.1%)
In union	137 (56.2%)	92 (61.3%)	45 (47.9%)
**Year of study n (%)**			
Year 1	44 (18.0%)	25 (16.7%)	19 (20.2%)
Year 2	114 (46.7%)	69 (46.0%)	45 (47.9%)
Year 3	79 (32.4%)	53 (35.3%)	26 (27.7%)
Year 4	7 (2.9%)	3 (2.0%)	4 (4.3%)
**Parental status n (%)**			
Yes	109 (44.7%)	37 (39.4%)	72 (48.0%)
No	135 (55.3%)	57 (60.6%)	78 (52.0%)

### Differences in time use by gender

On average, men and women reported a median of 6 days per week of paid work; the difference was not significant (median coefficient = 0.0, 95% CI: −0.8, 0.8). While it appears that women spend more time caring for household members (median = 3 vs 2 h/day) and less time on learning and studying activities (median difference = 9 h/week: 95% CI: −23.1, 5.1) compared to men, these differences were not statistically significant. Yet for unpaid domestic work, the median difference was 1 h/day and statistically significant (95% CI: 0.6, 1.4). Compared with women, men spent significantly more time on leisure activities (median = 7 vs 4 h/week, 95% CI: −5.3, −0.8), and the difference remained significant after adjusting for marital and parental status (Table 3).

**Table 3.** Gendered differences in paid and unpaid time.

Variable	Women Median (IQR)	Men Median (IQR)	Crude median coefficient (95% CI)	Adjusted median coefficient* (95% CI)	P-value
Paid work (days/week)	6.0 (2.0)	6.0 (2.0)	0.0 (−0.8, 0.8)	–	0.900
Time on unpaid care for household members (h/day)	3.0 (6.0)	2.0 (6.0)	1.0 (−0.7, 2.7)	–	0.270
Time on unpaid domestic work (h/day)	2.0 (1.5)	1.0 (1.0)	**1.0 (0.6, 1.4)**	**1.0 (0.6, 1.4)**	**0.000**
Time learning and studying (h/week)	15.0 (40.0)	24.0 (50.5)	−9.0 (−23.1,5.1)	–	0.240
Time on leisure (h/week)	4.0 (6.0)	7.0 (13)	**-3.0 (−5.3, −0.8)**	-**4.0 (−6.1, −1.9)**	**0.000**
Time on resting/sleeping (h/week)	40 (8)	35 (14)	**5 (1.3, 8.7)**	**5 (1.3, 8.7)**	**0.000**

*Adjusted for age, marital status and parental status.

### Median time use differences by gender, stratified by parental status

When stratified by parental status, women with children reported spending half a day more on paid work (95% CI: 0.2,1.8) and an additional hour on unpaid domestic work (95% CI: 0.4,1.6) compared to men with children. Differences in the other outcomes were not significant, even if women with children spent 1.5 more hours per day taking care of household members than men with children and 3 h less on leisure per week. Men with children appear to spend twice as much time studying as women with children (24 h/week vs. 12 h/week), although this difference was not statistically significant (Table 4). Among those without children, women spend an additional hour on unpaid domestic work (95% CI: 0.6,1.5) and 7 h more per week resting (95% CI: 3.0,11.0).

**Table 4.** Time use by gender stratified by parental status.

	Children		No Children		
Variable	Median (IQR)	Coefficient (95% CI)	Median (IQR)	Coefficient (95%CI)	P-value
**Paid work** (days/week)					
Men	6.5 (2.0)		6.0 (2.0)		
Women	7.0 (2.0)	**1.0 (0.2,1.8)**	6.0 (2.0)	0 (−0.9, 0.9)	1.00
**Unpaid domestic work** (h/day)					
Men	1 (1.0)		1 (1.0)		
Women	2 (2.0)	**1 (0.4,1.6)**	2 (1.0)	**1 (0.6,1.5)**	**0.000**
**Care for household members** (h/day)					
Men	2.5 (5)		2.0 (6)		
Women	4.0 (6)	1 (−0.8,2.8)	3.0 (6)	1 (−1.3,3.3)	0.400
**Studying** (h/week)					
Men	24 (50)		20 (51)		
Women	12 (34)	−12(−30.9,6.9)	20.5 (46)	1 (−1.3,3.3)	0.200
**Leisure** (h/week)					
Men	7 (10)		8.5 (17)		
Women	4 (5)	−3 (−6.4,0.4)	5 (7)	−4 (−8.1,0.1)	0.100
**Resting/sleeping (**h/week)					
Men	35 (14)		35 (14)		
Women	35 (8)	0 (−5.1,5.1)	42 (12)	**7 (3.0,11.0)**	**0.000**

Adjusted by age and marital status.

## Discussion

This study highlights gendered differences in time use among medical doctors in specialist training in Uganda. This could have important implications for study completion, career progression, and workforce management. After adjusting for age, marital and parental status, women reported spending significantly more time on unpaid domestic activities, while men reported spending more time on leisure. In regard to reported time spent on paid work, no significant gender differences were observed. Parenting impacted time use; women with children spent more time on paid work and on unpaid domestic work than men with children.

Our findings differ slightly from studies conducted in both high- and low-income countries, where, compared to women, men, regardless of their marital status and age, reported spending significantly more time in paid employment [3,45,46]. In this hospital study setting in Uganda, the similarity in reported time spent on paid work by men and women may reflect the structured demands of medical specialist training whereby, regardless of gender, doctors are required to spend specified hours in paid employment. It is important to highlight that among those with children, women spend significantly more time in paid employment than men with children. This is interesting to highlight because it challenges the stereotype that when women have children, they are less invested and dedicated.

Our findings are in line with other studies that show that women spend more time in unpaid domestic work [47–49]. Women doctors in this study reported spending almost one additional hour per day on unpaid domestic work, such as cooking and cleaning, even after adjusting for age, marital, and parental status. This gender difference in unpaid domestic work was significant for both those with and without children. This can be explained by traditional gender norms that place the responsibility for domestic tasks primarily on women [2].

Men, on the other hand, reported spending significantly more time on leisure activities even after adjusting for age, marital and parental status. This disparity can be attributed to the unequal distribution of unpaid domestic work and care activities. Similar patterns have been reported in other time use studies whereby unpaid domestic work encroaches on women’s time for rest and leisure [45,50]. However, among those with children, there were no significant differences between men and women in relation to leisure time or resting time. On the contrary, among those without children, women reported spending significantly more time resting or sleeping compared with their male counterparts, suggesting that parenting may influence time-use priorities.

Women reported spending more time than men on caregiving for household members, including young children, the ill, the elderly, and those with disabilities. Although these differences were not statistically significant, a noticeable trend was observed, which warrants verification through further studies with larger sample sizes. These findings are indeed consistent with prior studies showing that women, irrespective of income level or family status, shoulder a disproportionate share of domestic and caregiving responsibilities, especially in contexts lacking formal care structures [51,52].

Women spend a median of 9 h less per week learning and studying than men, and among the group with children, these differences increased to 12 h. Again, these differences were not statistically significant, which warrants verification with larger studies, but the trend was also noticeable. This resonates with concerns that women’s educational pursuits are often constrained by competing domestic activities and caregiving responsibilities. In the health workforce, women doctors’ reduced times for study and learning have long-term implications for skill development and career advancement. For instance, in Eastern Uganda, male health workers were more likely to participate in long-term training programs (averaging 20 months) compared to their female counterparts, who typically attended shorter training courses (averaging 10 months) [9]. These differences were attributed to gendered family responsibilities for men and women as dictated by cultural norms in many contexts [9,53].

According to the WHO Global Health Observatory, Uganda reported 71.5% male doctors and only 28.5% female doctors [54]. The gender distribution of our sample follows a similar pattern, with more men (61%) than women (38%) participating in our study and a higher proportion of men were married. This could be due to the fact that, even when men have partners and children, it is ‘easier’ for them to access higher education compared to their female counterparts who often take on a larger share of household and caregiving responsibilities. The higher population of men in specialty training in this study is indicative of the higher population of male doctors in Uganda, which shows that there are disparities in access to long-term education opportunities, but also indicates disproportionate access to medical training, seen all the way from undergraduate education. These systemic disparities continue to disadvantage females from entering, thriving and growing in the medical profession [55,56].

We also note several limitations. The findings should be interpreted in light of the following limitations. First, the study used self-reported data, which may be subject to recall bias and under- or over-reporting of time estimates. We considered self-declared information as reflecting the hours participants spend on activities and that this can be influenced by recall bias and even by gendered expectations (for example, women reporting dedicating more time to taking care of household members and doing unpaid domestic work because this is what society expects them to do). However, the methodology to measure time use through self-declared information has been evaluated for its robustness [57]. Second, we acknowledge that the study did not account for other potential confounding variables such as socio-economic status or cultural factors that could influence time-use patterns. Third, the study population was not representative of medical practitioners more generally in Uganda or elsewhere since it was conducted at one university. Medical doctors in specialist training typically work longer hours than established practitioners. Fourth, because the study was conducted in a single public university the findings may not be generalizable to other universities or settings. However, it is important to notice that Makerere is the largest medical school in the country and the only one with all specialties. Fifth, the study did not account for simultaneous activities and all activities that are done by an individual. Sixth, the study did not conduct a formal analysis of internal consistency across linked variables. Future studies could consider exploring internal consistency for linked variables. Finally, the gender stereotypes may be reinforced within our study since all confounders were not included in the analysis, leading to a biased narrative of time spent on paid work, unpaid work and leisure.

## Conclusion

Female medical doctors in specialist training reported spending significantly more time on unpaid domestic work and care activities, while their male counterparts reported spending more time on study and leisure activities. These findings provide a platform for further research in gendered time use in different health workforce settings.

Our findings have some implications. This study contributes to the growing body of evidence on how gendered time use shapes the daily lives of medical practitioners in specialist training. The findings underscore the need for gender-responsive policies that address the sorts of inequalities highlighted here. An example includes flexible work arrangements for both male and female health workers. There is a need for managers to incorporate gender-sensitive strategies in health workforce planning and development to promote equitable access to training and career development. Future research could explore the influence of factors such as employment type and socio-economic status and also look at the longer-term impacts on women’s career progression and well-being.

## Supplementary Material

STROBE_checklist_Gendered_time_use.doc

## Data Availability

The data for the study are available in Zenodo at https://doi.org/10.5281/zenodo.16941211 and can also be shared upon reasonable request.
