# Supplementary material for: Gendered time use among specializing medical doctors at Makerere University, Uganda: a cross-sectional study
Source: Glob Health Action. 2026 Mar 3;19(1):2636410. doi: 10.1080/16549716.2026.2636410 (PMC12958386; doi:10.1080/16549716.2026.2636410)
Supplement: STROBE_checklist_Gendered_time_use.doc [file ZGHA_A_2636410_SM7805.doc]

STROBE Statement—for a study titled, “**Gendered time use among specializing medical doctors at Makerere University, Uganda: a cross-sectional study”**

| **Item** | Item No | STROBE Recommendation | Study responses |
| --- | --- | --- | --- |
| **Title and abstract** | 1 | (*a*) Indicate the study’s design with a commonly used term in the title or the abstract | Abstract clearly states that a cross-sectional study design was used. |
|  |  | (*b*) Provide in the abstract an informative and balanced summary of what was done and what was found | The abstract provides an informative summary of what was done. The study design is stated, and a summary of the objectives, methods, and findings is given. |
| Introduction | | |  |
| Background/rationale | 2 | Explain the scientific background and rationale for the investigation being reported | The background highlights gendered time use and its impact on career advancement in Uganda’s medical workforce |
| Objectives | 3 | State specific objectives, including any prespecified hypotheses | 1. To analyze gendered differences in time use for paid and unpaid activities  2. To assess whether parenting influences time use. |
| Methods | | |  |
| Study design | 4 | Present key elements of study design early in the paper | Cross-sectional design as stated in methods section |
| Setting | 5 | Describe the setting, locations, and relevant dates, including periods of recruitment, exposure, follow-up, and data collection | Makerere University School of Medicine, Uganda, in 2024 |
| Participants | 6 | (*a*) Give the eligibility criteria, and the sources and methods of selection of participants | Graduate medical doctors in speciality training at Makerere University School of Medicine who accepted to participate in the study voluntarily. |
| Variables | 7 | Clearly define all outcomes, exposures, predictors, potential confounders, and effect modifiers. Give diagnostic criteria, if applicable | Time spent on paid work, unpaid domestic/care work, study, leisure; parenting status as exposure |
| Data sources/ measurement | 8* | For each variable of interest, give sources of data and details of methods of assessment (measurement). Describe comparability of assessment methods if there is more than one group | Self-administered questionnaire; self-reported time use across activities were the main sources of data. |
| Bias | 9 | Describe any efforts to address potential sources of bias | Recall bias about the time spent on different activities and self-reporting limitations were addressed by including questions to check responses given. |
| Study size | 10 | Explain how the study size was arrived at | Based on available cohort; response rate was 66% |
| Quantitative variables | 11 | Explain how quantitative variables were handled in the analyses. If applicable, describe which groupings were chosen and why | Time use analyzed via quantile regression; medians and confidence intervals reported. |
| Statistical methods | 12 | (*a*) Describe all statistical methods, including those used to control for confounding | Quantile regression used; subgroup analysis by gender and parenting status |
|  |  | (*b*) Describe any methods used to examine subgroups and interactions | N/A |
|  |  | (*c*) Explain how missing data were addressed | There were no missing variables |
|  |  | (*d*) If applicable, describe analytical methods taking account of sampling strategy |  |
|  |  | (*e*) Describe any sensitivity analyses |  |
| Results | | |  |
| Participants | 13* | (a) Report numbers of individuals at each stage of study—eg numbers potentially eligible, examined for eligibility, confirmed eligible, included in the study, completing follow-up, and analysed | 244 participants were included in this study out of the 369 graduate medical doctors who were invited to participate. |
|  |  | (b) Give reasons for non-participation at each stage | Reasons for unavailability included hospital commitments (in theatres or wards), getting a gap year, and declining to provide consent. |
|  |  | (c) Consider use of a flow diagram |  |
| Descriptive data | 14* | (a) Give characteristics of study participants (eg demographic, clinical, social) and information on exposures and potential confounders | There were three dichotomized independent variables: (i)*gender* (female vs male); (ii) *marital status* (in union vs not in union), and (iii) *parental status* (have children vs do not have children) that were included in the study |
|  |  | (b) Indicate number of participants with missing data for each variable of interest | N/A |
| Outcome data | 15* | Report numbers of outcome events or summary measures | Time use differences reported with medians and 95% CIs |
| Main results | 16 | (*a*) Give unadjusted estimates and, if applicable, confounder-adjusted estimates and their precision (eg, 95% confidence interval). Make clear which confounders were adjusted for and why they were included | Gender and parenting differences in time use were reported in the unadjusted and adjusted estimates during the analysis. |
|  |  | (*b*) Report category boundaries when continuous variables were categorized | We had age as our continuous variable and was categorised into two categories that is 25-34 and 35-50 |
|  |  | (*c*) If relevant, consider translating estimates of relative risk into absolute risk for a meaningful time period | N/A |
| Other analyses | 17 | Report other analyses done—eg analyses of subgroups and interactions, and sensitivity analyses | Parenting status was analyzed as subgroup |
| Discussion | | |  |
| Key results | 18 | Summarise key results with reference to study objectives | Gender imbalance in time use and its implications for career progression |
| Limitations | 19 | Discuss limitations of the study, taking into account sources of potential bias or imprecision. Discuss both direction and magnitude of any potential bias | First, the study used self-reported data, which may be subject to recall bias and under- or over-reporting of time estimates.  The study did not account for other potential confounding variables such as socio-economic status or cultural factors that could influence time-use patterns.  The study population was not representative of medical practitioners more generally in Uganda or elsewhere  The study did not account for simultaneous activities and all activities that are done by an individual. |
| Interpretation | 20 | Give a cautious overall interpretation of results considering objectives, limitations, multiplicity of analyses, results from similar studies, and other relevant evidence | Female medical doctors in specialist training reported spending significantly more time on unpaid domestic work and care activities than their male counterparts, who reported spending more time on study and leisure activities. The findings provide a platform for further research in gendered time use in different health workforce settings. |
| Generalisability | 21 | Discuss the generalisability (external validity) of the study results | The study was conducted in a single public university the findings may not be generalizable to other universities or settings. |
| Other information | | |  |
| Funding | 22 | Give the source of funding and the role of the funders for the present study and, if applicable, for the original study on which the present article is based | There was no funding attached to this this study. However, Makerere University School of Public Health contributed some funds towards data collection. |

*Give information separately for exposed and unexposed groups.

**Note:** An Explanation and Elaboration article discusses each checklist item and gives methodological background and published examples of transparent reporting. The STROBE checklist is best used in conjunction with this article (freely available on the Web sites of PLoS Medicine at http://www.plosmedicine.org/, Annals of Internal Medicine at http://www.annals.org/, and Epidemiology at http://www.epidem.com/). Information on the STROBE Initiative is available at www.strobe-statement.org.
